# Style and influencing factors of tutors-postgraduates’ interactions in Chinese medical colleges: a cross-sectional survey in Heilongjiang Province

**DOI:** 10.1186/s12909-023-04291-4

**Published:** 2023-05-02

**Authors:** Mingsi Wang, Yanping Wang, Ming Fang, Shue Zhang, Yilan Li, Depin Cao, Yan Jin

**Affiliations:** 1 Graduate School of Harbin Medical University, 157# Bao Jian Road, Harbin, 150081 China; 2 Department of Health Economics, College of Health Management of Harbin Medical University, 157# Bao Jian Road, Harbin, 150081 China; 3 Department of Health Management, School of Health Management, Harbin Medical University, 157# Bao Jian Road, Harbin, 150081 China; 4 Department of Cardiology, the Second Affiliated Hospital of Harbin Medical University, 148# Bao Jian Road, Harbin, 150000 China

**Keywords:** Analysis, Influencing Factors, Logistic Regression, Medical College, Postgraduate Education, Tutor-Postgraduate Interaction

## Abstract

**Objectives:**

This study assesses the style of tutor-postgraduate interactions in Chinese medical colleges and explores the association between postgraduates’ demographic factors and tutors’ demographic characteristics.

**Methods:**

With the stratified sampling method, a cross-sectional online survey was used. A total of 813 medical postgraduates were recruited as participants, with an effective response rate of 85.49%. The two dimensions of “Professional Ability Interaction” and “Comprehensive Cultivation Interaction” in the self-developed “Instructor-Graduate Interaction Scale for Medical Colleges” were used as dependent variables. And tutors’ demographic characteristics and postgraduates’ demographic characteristics were taken as independent variables. Logistic regression analysis was used to explore the influencing factors of Tutor-Postgraduates Interactions in medical colleges.

**Results:**

The Tutor-Postgraduates Interaction scale consists of 14 items from the two dimensions of “Professional Ability Interaction” and “Comprehensive Cultivation Interaction”. The results of the logistic regression analysis show the reasons for selecting the mentor students (industry recognition, the tutor’s research direction, charm in attracting mentors, and recommendations for mentor selection); student to mentor satisfaction; student to study life satisfaction; and regular academic seminars. Indirect guidance and a high postgraduate grade high are the protective factors of interaction between tutors and postgraduates of medical colleges and universities postgraduates. Older mentors and more graduate tutors are the risk factors for Tutor-Postgraduates Interaction in medical colleges (*P* < 0.05).

**Conclusion:**

The current study proposes that managers should pay more attention to the double-track promotion of “Professional Ability Interaction” and “Comprehensive Cultivation Interaction”. We should not only pay attention to the cultivation of postgraduates’ professional ability but also pay more attention to the comprehensive cultivation including postgraduates’ mental and psychological aspects. The interaction between tutors and postgraduates in medical colleges is generally good, but much attention should be given to the dual-track promotion mentioned above. Regular academic seminars play an important role in the process of postgraduate training. The research findings, including the influencing factors regarding tutor-postgraduate interactions, the Professional Ability Interaction and Comprehensive Cultivation Interaction, are very informative and can contribute to strategies for postgraduate management systems that enhance this relationship.

## Background

Postgraduate education is a post-undergraduate higher education aimed at obtaining a master’s degree or above, with scientific research as the main means of learning. Through postgraduate education and training, students can have a wider range of knowledge, a deeper accumulation of knowledge, and more professional research content. Postgraduate education can cultivate a student’s ability to innovate and solve problems independently. An objective of postgraduate medical education is to help postgraduates advance their careers as health care professionals and stay up-to-date on the latest advancements in medical research and clinical practice. The postgraduate medical education (PGME) teaching mode and postgraduate-tutor interaction style differ substantially within different cultural settings [1]. For example, postgraduate medical education in the Netherlands has adopted competency-based education [2]. In China, the postgraduates’ tutor responsibility system has been adopted in postgraduate education, who is the principal supervisor in charge of academic training and postgraduate life. Compared to Western countries, Chinese postgraduates tutor bear the responsibility of moral transmission during academic training [3]. Moreover, the Ministry of Education, the most authoritative institution in China’s education, issued the “Opinions of the Ministry of Education on the full implementation of the duties of morality education of the postgraduate tutor” in January 2018 (Teaching and Research [2018] No.1). The postgraduate tutor’ basic quality of postgraduate education, the evaluation and incentive mechanism for postgraduate tutors, and the duties of morality education of postgraduates be conscientiously stressed in mentioned above standard. Furthermore, the tutor system in China has deepened and is continuously being perfected. Postgraduate tutors have been given more power and have assumed more responsibilities at the same time. At the 33rd meeting of the Academic Degree Committee of the State Council in 2017, Vice Premier Liu Yandong pointed out that the tutor was the first person responsible for postgraduate education and the first person responsible for the task of cultivating good moral character [4]. Therefore, experienced training in the postgraduate learning stage not only includes the cultivation of postgraduate research competency and professional skills, but also including responsible for enhancing professional spirit, moral sentiment, and so on [3]. Therefore, tutors are one of the decisive factors in the quality of postgraduate education [5].

In addition, the interaction style between postgraduates and their tutors is also a key factor affecting the quality of postgraduate academic training. Studies have shown that communications and interactions between postgraduates and their tutors affect the cultivation mode, thus affecting the quality of postgraduate academic performance [6]. In addition, previous studies revealed that effective tutor-student interactions have a positive impact on learning engagement, academic performance, and academic achievement [6, 7]. Usually, the postgraduate-tutor interaction style includes core elements of training and ongoing development of postgraduate education [8]. The existing studies on the interaction between postgraduates and their tutors in China mainly include three categories. Based on different classification criteria, some scholars divided postgraduate tutors’ type into the laissez-faire type, the free-range type, the director type, and the contractual type according to the postgraduate tutors’ strict degree toward postgraduate [9]. Secondly, according to the alienation of the relationship between postgraduates and their tutors, some studies divide the relationship between postgraduates and their tutors into “pure teacher-student type”, “boss-employee type”, “teacher-friend type” and so on [10]. Third, according to the teaching methods and styles of postgraduate tutors, some studies concluded the relationship between postgraduates and their tutors is “tight”, “democratic”, “controlling” and “loose” [11]. Furthermore, the interaction style between medical postgraduates’ tutors and their students is markedly different from that of other majors. Medical postgraduates’ tutors are responsible for guiding and instructing postgraduates in their professional theory study, clinical skills, subject research, daily life, and the formation and development of the three outlooks [4]. In this process of postgraduate training, the harmonious interaction between tutors and postgraduates contributes to the construction of an effective teaching atmosphere, improves communication quality between tutors and postgraduates, and enhances academic progress in postgraduate training [12]. However, the roles of medical postgraduate tutors in China are relatively diverse, undertaking many tasks, such as medical professional services, medical postgraduate education, and medical scientific research development. This leads to less communication and interaction between tutors and postgraduates in medical colleges both in terms of frequency and quality. A heavy workload for tutors is not conducive to the improvement of the quality of medical postgraduate training and may potentially increase medical risks in future postgraduate practice. Few studies have paid attention to this interaction between medical postgraduates and tutors. Therefore, it is of great significance for the quality of postgraduate education to explore the interaction style between postgraduate medical tutors and their students in the context of Chinese culture and help build a harmonious interaction mode between tutors and postgraduates [7]. Therefore, this study attempts to classify the interaction types of medical postgraduates’ tutors and their students. And further, study the influencing factors of the interaction between medical postgraduates’ tutors and their students.

### Aims

In summary, this study aims to (1) identify the style of the interaction between postgraduate tutors and postgraduates in medical colleges in China, and (2) identify the influencing factors for different tutor-postgraduate interactions in medical colleges based on Chinese medical postgraduates’ own perspectives.

## Methods

### Subjects and procedures

A cross-sectional online survey was conducted using the stratified sampling method. Sampling was stratified according to different grades, and questionnaire surveys were conducted. The research was conducted following the guidelines of the Declaration of Helsinki and approved by the ethics committee of the Institutional Review Board at Harbin Medical University (ECHMU). All subjects provided informed consent to participate in this study. A total of 951 questionnaires were collected, and 813 valid questionnaires were obtained. Questionnaires with short responses were excluded. The effective rate of the questionnaire was 85.49%. The questionnaire survey was conducted in the form of online research and published through the Questionnaire Star network platform. The respondents could fill in the questions via their mobile phone, WeChat, or website link. The subjects were postgraduates in medical colleges and universities in China. Demographic information of both students and their tutors was collected through a self-administered questionnaire distributed to the students. The questionnaire was published in three ways. First, it was directly distributed through the postgraduate department of the College of Medical Colleges and Universities in Heilongjiang Province. Second, it was distributed among members and clients of the research group. A random red envelope was sent through the platform to encourage respondents to fill in the questionnaires seriously. The quality control test was set at the end of the questionnaire. The quality control process was carried out by a four-member questionnaire quality screening team, with two teams and double-checking, as a way to avoid questionnaire quality bias caused by subjective factors.

### Measures

#### Measurement of postgraduates’ demographic characteristics

In this study, ten demographic variables were adopted, including (1) gender, (2) grade, (3) admission methods of postgraduates (recommended exemption/national unified examination/transition stage[‘5 + 3’ integrated training of clinical medicine]), (4) source of postgraduate (Town/Non town), (5) major (clinical postgraduate/nonclinical postgraduate), (6) only child or no, (7) annual family income, (8) more than one year of formal working experience, (9) reasons for postgraduates to choose their current major (including their own or parents interests; tutors, seniors or other people made suggestions; good employment prospects; applying for the professional level of the tutor; higher probability of admission to graduate entrance examination; postgraduates can only choose this major because of the major limitation of the undergraduate major or target job; postgraduates were transferred to their current majors; and/or other reasons), and (10) the reasons postgraduates choose their current tutors (including the academic level of the tutor is recognized by his or her peers in the research field; the tutor’s administrative position or academic status is high, which is conducive to the individual’s future career development; the tutor’s personal charm; parents, tutors, seniors or other people’s suggestions; postgraduates are interested in the directions of the tutors’ research; higher probability of admission to graduates entrance examination; fewer postgraduates apply for this tutor and it is easier to get in; postgraduates were transferred to their current tutors; and/or other reasons).

#### Measurement of tutors’ demographic characteristics

In this study, ten demographic variables of tutors were adopted, including gender, age, professional title, administrative position, number of tutors directing all postgraduates, tutor style, teaching style, research group, and graduate numbers. Each postgraduate tutor pays different attention to affairs and academic research, thus forming different styles of tutors. In this study, the tutor style is a self-made scale, including 6 items. The self-made scale contains two dimensions, named “tutor’s concern for academic research” and “tutor’s concern for the work of affairs”. Exploratory factor analysis showed that the weight of two common factors in the scale could explain 81.252% of all items, and the scale had good construct validity. The Cronbach’s α coefficient of the whole scale was 0.866. The scale passed the reliability and validity test. Some of the questions are as follows: “Your tutor has devoted a lot of time to scientific research”, and “Your tutor attaches great importance to his professional (clinical, teaching, administrative) work”. The teaching style was categorized as assigned postgraduate tutor or not. Postgraduates’ satisfaction with their tutors was categorized as Very dissatisfied/Not satisfied/General/Satisfied/Very satisfied. Postgraduates’ satisfaction with graduate studies and academic life was classified as Very dissatisfied/Not satisfied/General/Satisfied/Very satisfied.

#### Measurement of interaction scale between tutors and postgraduates in medical colleges

An extensive review of the literature was conducted. The results of this were combined with the interview results with the graduate management experts, graduate tutors, and other relevant insiders, and an analysis was conducted. Drawing on the scholarly work of Wang Qian, Meng Yi, and Zhang Rui, a tool for measuring the interaction between tutors and postgraduates in medical colleges was developed, entitled the “interaction scale between supervisors and postgraduates in medical colleges”. The scale contains 14 items, using Likert 5 (1 point is “very inconsistent”, 2 points is “comparatively inconsistent”, 3 points is “general”, 4 points is “comparatively consistent”, and 5 points is “very consistent”) as the criteria. The higher the score is, the higher the level of interaction between the tutor and the postgraduate.

### Statistical analysis

The valid questionnaire data was imported into Microsoft Office Excel software and SPSS 19.0 software from the Questionnaire Star platform for statistical analysis, descriptive statistical analysis, internal consistency reliability coefficient (Cronbach’s alpha), logistic regression analysis, etc. The test level was *a* = 0.05.

## Results

### Demographic information for samples

A total of 813 participants were surveyed, all of whom were postgraduates from medical colleges (Table 1). Most of the postgraduates were from Harbin Medical University, Mudanjiang Medical University, and Jiamusi University. In terms of gender distribution, 216 males, accounted for 26.6% of the total number. There were 597 females, accounting for 73.4% of the total. In their respective grades, there were 278 first grade masters, accounting for 34.2% of the total. In admission methods of postgraduates, a total of 728 postgraduates were enrolled through the national unified examination, accounting for 89.5% of the total. From the source of postgraduates, there were 506 postgraduates from town, accounting for 62.2% of the total. In terms of degree types, the number of professional degrees was 435, accounting for 53.5% of the total.

**Table 1:** Basic information of the participants

Demographic Characteristics	Category	Number (N)	Constituent ratio (%)
Gender	Male	216	26.6
	Female	597	73.4
Grade	First Grade Master	278	34.2
	Second Grade Master	276	33.9
	Third Grade Master	259	31.9
Admission methods of postgraduates	Recommended exemption	43	5.3
	National unified examination	728	89.5
	Transition stage	42	5.2
Source of postgraduates	Town	506	62.2
	Non town	307	37.8
Major	Clinical Specialty	490	60.3
	Non-clinical specialty	323	39.7
Degree type	Academic degree	378	46.5
	Professional degree	435	53.5
Duties during postgraduate study	No duty	559	68.8
	Now or once a postgraduate cadre	254	31.2
Only-child or no	Yes	426	52.4
	No	387	47.6
Annual family income	< 20,000 Yuan	222	27.3
	20,000–50,000 Yuan	242	29.8
	50,000-100,000 Yuan	214	26.3
	100,000-200,000Yuan	98	12.1
	> 200,000 Yuan	37	4.6
Have more than one year of formal working experience	Yes	101	12.4
	No	712	87.6

The basic situation of the tutors and the basic situation of the tutors guiding postgraduates were also measured (Table 2). In terms of gender, about 361 males, accounted for 44.4% of the total. 452 females, accounted for 55.6% of the total. In the age distribution of postgraduate tutors, there were 365 tutors between 51 and 60 years old, accounting for 44.9% of the total number. In terms of the titles of tutors, there were 637 professors, accounting for 78.4% of the total. In terms of the number of all postgraduates supervised by the tutor of the survey object, there were 400 tutors of 1–5 postgraduates, accounting for 49.2% of the total number.

**Table 2:** Basic situation of participants’ target tutors

Demographic Characteristics	Category	Number (N)	Constituent ratio (%)
Gender of tutors	Male	361	44.4
	Female	452	55.6
Age of tutors	31–40	106	13.0
	41–50	314	38.6
	51–60	365	44.9
	> 61	28	3.4
Tutors’ positional titles	Professor	637	78.4
	Associate professor	176	21.6
Has the tutor assigned a young teacher or senior postgraduates to give you daily guidance?	Yes	515	63.3
	No	298	36.7
Is the tutor a clinician?	Yes	469	57.7
	No	344	42.3
Administrative position of tutor	No administrative position	174	21.4
	Director (deputy) of a teaching and research department or director (deputy) of a department	452	55.6
	College (deputy) dean or middle-level cadres	132	16.2
	School leadership or higher positions	55	6.8
Number of tutors directing all postgraduates (including PhD and Master’s degrees in all grades)	1–5	400	49.2
	6–10	203	25.0
	11–15	108	13.3
	16–20	63	7.7
	> 21	39	4.8
Whether there are regular academic seminars?	Yes	621	76.4
	No	192	23.6

### Exploratory factor analysis and reliability analysis of the questionnaire between tutors and postgraduates in medical colleges and universities

To ensure the overall reliability and validity of the questionnaire, the internal consistency reliability test was used to test its reliability and validity [13]. The results showed that the Cronbach α coefficient was 0.958, which is higher than the commonly used psychometric standard of 0.700, and has high reliability [14, 15]. At the same time, the scale has good structural validity through factor analysis.

Fourteen items were analyzed by exploratory factor analysis (Table 3). Bartlett’s spherical test coefficient was 0.000, which reached an obvious level. The KMO coefficient was 0.951, approaching 1, which indicates that the variables of the scale meet the requirements of exploratory factor analysis [16, 17]. The principal component analysis method was used to extract the main factors from 14 items according to the condition that the eigenvalue is greater than 1 [18, 19]. The maximum variance method was used to rotate the factor load matrix [20]. The results showed that exploratory factor analysis of principal component analysis extracts two common factors, and the weights of two common factors can explain 74.273% of all items, which can reflect most of the information of indicators [21]. It can be confirmed that the “Instructor-Graduate Interaction Scale for Medical Colleges” has good structural validity.

**Table 3:** Exploratory factor analysis factor component matrix of instructor-graduate interaction scale in medical colleges

Item	Factor loading	
	Professional Ability Interaction	Comprehensive Cultivation Interaction
1. The tutor is very strict with your scientific research	0.861	
2. The tutor always checks your research work	0.840	
3. The tutor sets goals or deadline for your research work	0.815	
4. The tutor often cares about all kinds of special situations you may encounter in your work (scientific research or clinical)	0.773	
5. The tutor has strong scientific research guidance ability	0.750	
6. Whenever you submit your research results, the tutor will reply as soon as possible	0.659	
7. The tutor often shares his work (scientific research or clinical) experience with you	0.629	
8. The tutor often chats with you		0.849
9. The tutor has cared about your personal emotional problems		0.838
10. The tutor cares about your future job intention		0.754
11. The tutor often cares about whether there are difficulties in your life		0.752
12. You have learned a lot from your tutor about how to deal with interpersonal relation		0.718
13. The tutor pays great attention to the improvement of your comprehensive ability		0.696
14. Through the guidance of your tutor, your ability to face the job in the future has been greatly improved		0.660

These two dimensions were named “Professional Ability Interaction” and “Comprehensive Cultivation Interaction”. “Professional Ability Interaction” refers to the interaction between tutors and postgraduates related to the scope of scientific research and academic discussion, which focuses on the teaching behavior of tutors toward postgraduate academic and clinical ability. “Comprehensive cultivation interaction” refers to the interaction between tutors and postgraduates, in addition to the necessary academic interactions, in terms of ideology, life, future development, and comprehensive quality training, such as the cultivation of mentors in postgraduate personal life and the solution of difficulties in postgraduate life. The Cronbach α coefficients of the two dimensions were 0.938 and 0.935, respectively, and the overall reliability of the scale was 0.958. Thus, the reliability coefficients of the “Tutor-Postgraduate Interaction Scale” and all dimensions of the scale were recorded at a high level, so the “Tutor-Postgraduate Interaction Scale” and all dimensions of the scale have good reliability [22].

### The status of interaction between tutors and postgraduates in medical colleges

The descriptive results showed that the overall score of the interaction between tutors and postgraduates in medical colleges was (3.82 ± 0.94), a high score indicating that the overall situation between tutor-postgraduate interaction is better, with the scores of Professional Ability Interaction being (3.91 ± 0.96) and the scores of Comprehensive Cultivation Interaction being (3.73 ± 1.03).

### Multivariate logistic regression analysis of influencing factors of interaction between tutors and postgraduates in medical colleges

To analyze the influencing factors of the interaction between tutors and postgraduates in medical colleges and universities, the two dimensions of “Professional Ability Interaction” and “Talent Cultivation interaction” of “Tutors-Postgraduates interaction” were reassigned and analyzed by multivariate logistic regression [23]. The two dimensions of “Professional Ability Interaction” and “Comprehensive Cultivation Interaction” after assignment were taken as dependent variables, and tutors’ demographic characteristics and postgraduates’ demographic characteristics were taken as independent variables.

The dimension of “Professional Ability Interaction” was reassigned (0 = low Professional Ability Interaction, 1 = high Professional Ability Interaction). After recoding, the factors related to tutor-postgraduate interaction were analyzed by multivariate logistic regression. Gender of postgraduates (Male = 1, Female = 2); Grade of postgraduates (First Grade Master = 1, Second Grade Master = 2, Third Grade Master = 3); Admission methods of postgraduates (Recommended exemption = 1, National unified examination = 2, Transition stage[‘5 + 3’ integrated training of clinical medicine] = 3); Source of postgraduate (Town = 1, Non town = 2); Major (Clinical Specialty = 1, Nonclinical specialty = 2); Only child or not (Yes = 1, No = 2); Family annual income (< 20,000 Yuan = 1, 20,000–50,000 Yuan = 2, 50,000-100,000 Yuan = 3, 100,000-200,000 Yuan = 4, > 200,000 Yuan = 5); Have more than one year of formal working experience (Yes = 1, No = 2); Reasons for postgraduates to choose their current major (Own interest = 1, Parents, teachers, seniors or other people suggest = 2, Good employment prospects = 3, Apply for the professional level of the tutor = 4, Higher probability of admission to graduates entrance examination = 5, Graduate students can only choose this major because of the major limitation of the postgraduate undergraduate major or target job = 6, Postgraduates were transferred to their current majors = 7, Other reasons = 8); Reasons why postgraduates choose their current tutors (The academic level of the tutor is recognized by his or her peers in the research field = 1, The tutor’s administrative position or academic status is high, which is conducive to the individual’s future career development = 2, The tutor’s personal charm = 3, Parents, teachers, seniors or other people’s suggestions = 4, Postgraduates are interested in the directions of the tutors’ research = 5, Higher probability of admission to graduates entrance examination = 6, Fewer students applied for this tutor and it was easier to get in = 7, Postgraduates were transferred to their current tutors = 8, Other reasons = 9); Gender of tutors (Male = 1, Female = 2); Age of tutors (31–40 = 1, 41–50 = 2, 51–60 = 3, > 61 = 4); Tutors’ positional titles (Professor = 1, Associate professor = 2); Administrative position of tutor (No administrative position = 1, Director[deputy] of a teaching and research department or director[deputy] of a department = 2, College[deputy] dean or middle-level cadres = 3, School leadership or higher positions = 4); Number of tutors directing all postgraduates (including PhD and Master’s degrees in all grades) (1–5 = 1, 6–10 = 2, 11–15 = 3, 16–20 = 4, > 21 = 5); Has the tutor assigned a young teacher or senior postgraduates to give you daily guidance? (yes = 1, no = 2); Whether there are regular academic seminars? (yes = 1, no = 2); postgraduates’ satisfaction with their tutors (very dissatisfied = 1, not satisfied = 2, general = 3, satisfied = 4, very satisfied = 5); postgraduates’ satisfaction with graduate studies and campus life (very dissatisfied = 1, not satisfied = 2, general = 3, satisfied = 4, very satisfied = 5).

#### Professional ability interaction—multivariate analysis

The odds ratio (OR) for variable X was adjusted for potential confounding factors. The results showed whether the selection of tutors was because they were recognized by the industry and their academic level (*Waldχ*^*2*^ = 4.555, *P* < 0.05, *OR* = 1.488, 95%*CI *1.033–2.143), whether they were interested in the research direction of tutors (*Waldχ*^*2*^ = 5.473, *P* < 0.05, *OR* = 1.577, 95%*CI *1.077–2.309), whether there were regular academic seminars (*Waldχ*^*2*^ = 29.795, *P* < 0.05, *OR* = 0.304, 95%*CI* 0.198–0.466), the age group of tutors (Wald*χ*^*2*^ = 12.236, *P* < 0.05, *OR* = 0.650, 95%*CI* 0.511–0.827), postgraduates’ satisfaction with tutors (*Waldχ*^*2*^ = 67.499, *P* < 0.05, *OR* = 2.986, 95%*CI* 2.300-3.876), and postgraduates’ satisfaction with their learning life (*Waldχ*^*2*^ = 6.953, *P* < 0.05, *OR* = 1.328, 95%*CI* 1.076–11.640). These six factors affect the mutual professionalism of tutors and postgraduates. The influence of the professional ability interaction level was statistically significant (*P* < 0.05). The overall model significance test *χ*^*2*^ = 331.770 (*P =* 0.00 < 0.01) reached the 0.01 significance level, while the Hosmer–Lemeshow test value was 10.490 (*P =* 0.232 > 0.05), which did not reach a significant level, indicating that the fitness of the regression model was ideal [24]. The assignment and results are shown in Table 4.

**Table 4:** Multivariate Logistic Regression Analysis of “Professional Ability Interaction” between tutors and postgraduates

Independent Variable	Assignment Situation	*B*	*SE*	*Wald*	*P*	*Adjusted OR*	95%*CI*
1. The academic level of the tutor is recognized by his or her peers in the research field	1 = unselected 2 = selected	0.397	0.186	4.555	0.033	1.488	1.033–2.143
2. Postgraduates are interested in the directions of the tutors’ research	1 = unselected 2 = selected	0.455	0.195	5.473	0.019	1.577	1.077–2.309
3. Whether there are regular academic seminars	1 = Yes, 2 = No	-1.190	0.218	29.795	0.000	0.304	0.198–0.466
4. Age group of tutors	1 = Age 30 and under 2 = 31–40 3 = 41–50 4 = 51–60 5 = Age 61 and over	-0.431	0.123	12.236	0.000	0.650	0.511–0.827
5. Postgraduates’ satisfaction with tutors	1 = Very dissatisfied 2 = Dissatisfied 3 = General feeling 4 = Satisfied 5 = Very satisfied	1.094	0.133	67.499	0.000	2.986	2.300-3.876
6. Postgraduates’ satisfaction with their learning life	1 = Very dissatisfied 2 = Dissatisfied 3 = General feeling 4 = Satisfied 5 = Very satisfied	0.284	0.108	6.953	0.008	1.328	1.076–11.640
Overall model fitness test: *X*^2^ = 331.770, Hosmer-Lemeshow test = 10.490							

#### Comprehensive cultivation interaction-multivariate analysis

The dimension of “Comprehensive Cultivation Interaction” is reassigned (0 = low Comprehensive Cultivation Interaction, 1 = high Comprehensive Cultivation Interaction). After recoding, the correlation factors of tutor-postgraduate interaction were analyzed by logistic regression. The results showed the grade of postgraduates (*Waldχ*^*2*^ = 8.007, *P* < 0.05, *OR* = 1.378, 95%*CI *0.293–0.709), whether the selection of tutors was attracted by their personal charm (*Waldχ*^*2*^ = 20.569, *P* < 0.05, *OR* = 2.367, 95%*CI *2.233–3.855), whether the selection of tutors was suggested by parents, tutors or classmates (*Waldχ*^*2*^ = 3.521, *P* < 0.05, *OR* = 1.496, 95%*CI *1.064–1.632), the number of postgraduates supervised by tutors (*Waldχ*^*2*^ = 18.889, *P* < 0.05, *OR* = 0.713, 95%*CI* 1.103–1.720), whether there were regular academic seminars (*Waldχ*^*2*^ = 12.149, *P* < 0.05, *OR* = 0.456, 95%*CI* 1.631–3.436), whether the tutor appoints young tutors or senior postgraduates to give daily guidance (*Waldχ*^*2*^ = 11.816, *P* < 0.05, *OR* = 0.518, 95%*CI* 0.982–2.279), postgraduates’ satisfaction with the tutor (*Waldχ*^*2*^ = 59.712, *P* < 0.05, *OR* = 2.934, 95%*CI *0.612–0.830) and postgraduates’ satisfaction with learning life (*Waldχ*^*2*^ = 6.998, *P* < 0.05, *OR* = 1.318, 95%*CI* 0.356–0.754) significantly affected the level of professional ability interaction between the tutors and postgraduates (*P* < 0.05). The overall model significance test *χ*^*2*^ = 344.305 (*P* = 0.00 < 0.01) reached the 0.01 significance level, while the Hosmer–Lemeshow test value was 7.783 (*P* = 0.455 > 0.05), which did not reach a significant level, indicating that the fitness of the regression model was ideal [25]. The assignment and results are shown in Table 5.

**Table 5:** Multivariate Logistic Regression Analysis of “Comprehensive Cultivation Interaction” between tutors and postgraduates

Independent Variable	Assignment Situation	*B*	*SE*	*Wald*	*P*	*Adjusted OR*	95%*CI*
1. The grade of postgraduates	1 = First Grade Master 2 = Second Grade Master 3 = Third Grade Master	0.320	0.113	8.007	0.005	1.378	0.293–0.709
2. The selection of tutors was attracted by their personal charm	1 = unselected 2 = selected	0.862	0.190	20.569	0.000	2.367	2.233–3.855
3. The selection of tutors was suggested by parents, tutors, seniors or other people	1 = unselected 2 = selected	0.403	0.215	3.521	0.061	1.496	1.064–1.632
4. The number of postgraduates supervised by tutors	1 = 1–5 2 = 6–10 3 = 11–15 4 = 16–20 5 = more than 21	-0.339	0.078	18.889	0.000	0.713	1.103–1.720
5. Whether there are regular academic seminars	1 = Yes 2 = No	-0.785	0.225	12.149	0.000	0.456	1.631–3.436
6. Has the tutor assigned a young teacher or senior postgraduates to give you daily guidance?	1 = Yes 2 = No	-0.657	0.191	11.816	0.001	0.518	0.982–2.279
7. Postgraduates’ satisfaction with tutors	1 = Very dissatisfied 2 = Dissatisfied 3 = General feeling 4 = Satisfied 5 = Very satisfied	1.076	0.139	59.712	0.000	2.934	0.612–0.830
8. Postgraduates’ satisfaction with their learning life	1 = Very dissatisfied 2 = Dissatisfied 3 = General feeling 4 = Satisfied 5 = Very satisfied	0.276	0.109	6.402	0.011	1.318	0.356–0.754
Overall model fitness test: *X*^*2*^ = 344.305, Hosmer-Lemeshow test = 7.783							

## Discussions

### The interaction between tutors and postgraduates in medical colleges

The Tutors-Postgraduates Interaction scale has good internal consistency and structural validity and is composed of 14 items divided into two dimensions: Professional Ability Interaction and Comprehensive Cultivation Interaction. The results show that the overall score of the interaction between tutors and postgraduates in medical colleges is (3.82 ± 0.94), and the score is higher than the median, indicating that the overall situation between tutors-postgraduates interaction is better. The study found that the scores of Professional Ability Interaction (3.91 ± 0.96) were slightly higher than the scores of Comprehensive Cultivation Interaction (3.73 ± 1.03). The results show that the interaction between medical postgraduate tutors and their students in China is more inclined toward the interaction of professional topics. It is worth mentioning that there are some differences in the interaction between domestic and foreign postgraduates and their tutors. Postgraduate tutors in European and American countries will give more freedom to their students and give more support and suggestions regarding research direction [26]. Postgraduate tutors encourage their students to actively participate in academic discussions and challenges during the postgraduates’ academic training. In addition, European and American tutors usually give medical postgraduates more space for independent thinking and critical thinking [26]. Postgraduates need to be responsible for their own academic direction and experimental design, as well as their own academic results. Therefore, the interaction between medical postgraduates and their tutors in European and American countries is more active, free, and open. Domestic postgraduate tutors pay more attention to cooperation with postgraduate tutors’ teams. Most of the postgraduates continue to carry out academic work with the projects of their tutors and senior teachers. Therefore, domestic postgraduates generally expect to establish a deeply close relationship with their tutors, and their academic attachment to tutors was higher than Western postgraduates. Noteworthy, it is equally important for postgraduates to train in comprehensive ability and professional ability. Therefore, this study suggests that medical education administrators should strengthen the dual-track of professional ability interaction and comprehensive cultivation interaction. That is to say, we should not only attach importance to the cultivation of postgraduates’ professional ability but also attach importance to the cultivation of postgraduates’ comprehensive ability such as spiritual psychology. What’s more, we should further pay attention to improving postgraduates’ ability of critical thinking and academic design, providing postgraduates enough independent thinking space, and cultivating medical talent with high professional skill levels and ethics.

### The influencing factors of “professional ability interaction” and “comprehensive cultivation interaction” between postgraduates and tutors in medical colleges

The results show that irregular academic seminars in teaching departments have a negative association with the interaction between tutors and postgraduates in medical universities, which is a risk factor for high-level Professional Ability Interaction and Comprehensive Cultivation Interaction between tutors and postgraduates. That is, there are regular academic seminars in the teaching department to promote deep interaction, academic discussion, and course discussion between tutors and postgraduates [27]. Moreover, academic seminars can increase the depth and breadth of academic communication between tutors and postgraduates and enhance the emotion between tutors and postgraduates. This finding suggests that regular academic seminars can, not only increase academic efficiency but also improve tutor management toward mentoring postgraduates.

The results show that postgraduates’ satisfaction with their tutors and their current learning life has a significant positive predictive effect on the interaction between tutors and postgraduates in medical colleges and universities and is a protective factor for the interaction between tutors and postgraduates with a high level of Professional Ability Interaction and Comprehensive Cultivation Interaction. The higher the degree of satisfaction of postgraduates with their mentors, the higher their recognition of them, and the more willing they are to communicate with them emotionally [28]. Especially when postgraduates are very satisfied with their studies and lifestyle during the postgraduate study, the conditions and cultural environment provided by universities for postgraduates are better. The recognition of mentors within schools also promotes positive interaction between tutors and postgraduates.

### The influencing factors of the “professional ability interaction” between tutors and postgraduates in medical universities

The results show that postgraduates believe that the academic level of tutors is recognized by the industry and this entails a certain level of academic popularity. Another reason why postgraduates choose tutors is their interest in the research direction of tutors, which has a significant positive predictive effect on the Professional Ability Interaction between tutors and postgraduates and is a protective factor for the Professional Ability Interaction between tutors and postgraduates at a high level. The reason may be that when postgraduates choose tutors, because their academic level of tutors is recognized by the industry and has a certain degree of academic popularity postgraduates will psychologically recognize the academic level of tutors because of the primary impression effect [29]. Postgraduates will take the initiative to ask questions of tutors to enhance their ability and learn from their professional knowledge, which promotes the interaction between tutors and postgraduates [30]. At the same time, the tutors are willing to impart their knowledge to postgraduates, which promotes the interaction between tutors and postgraduates, and, in turn, enhances their academic popularity and reputation. Most postgraduates choose topics similar to or related to their tutors. When postgraduates are interested in the research direction of tutors, they are also interested in their own research. In the process of scientific research, their subjective initiative plays a role, thus promoting the Professional Ability Interaction between tutors and postgraduates.

The results show that the older age group has a significant negative predictive effect on Professional Ability Interaction between tutors and postgraduates, and the older mentor is a risk factor for high-level Professional Ability Interaction between tutors and postgraduates. That is, the younger the tutor’s age, the higher the degree of Professional Ability Interaction between tutors and postgraduates [31]. The reason may be that the closer the age of tutors and postgraduates is, the smaller the generation gap will be and a more common language between tutors and postgraduates will be utilized.

### Analysis of the influencing factors of the “comprehensive cultivation interaction” between tutors and postgraduates in medical colleges and universities

The results show that senior postgraduates have a significant positive predictive effect on Comprehensive Cultivation Interaction between tutors and postgraduates, which is a protective factor for high-level Comprehensive Cultivation Interaction between tutors and postgraduates. That is, the higher the grade of postgraduates, the higher the degree of Comprehensive Cultivation Interaction between tutors and postgraduates. The reason may be that with time passing, the mentor’s understanding of postgraduates becomes increasingly more in-depth, and the temperament of the postgraduates, including ways of dealing with people, the world and shortcomings in his/her personality are increasingly understood. Thus, the mentor’s guidance on the ideological, emotional, and comprehensive qualities of postgraduates increases [32]. At the same time, with the rise of the importance of grades, postgraduates will face employment opportunities and enter society formally, and tutors will give more guidance regarding the future development and employment of postgraduates [33]. In summary, the interaction degree between senior postgraduates and tutors is high.

The results show that postgraduates choose tutors because of their personal attraction and the suggestion of parents, tutors, or classmates, which have a significant positive predictive effect on Comprehensive Cultivation Interaction between tutors and postgraduates and is the protective factor of high-level Comprehensive Cultivation Interaction between tutors and postgraduates. If postgraduates choose tutors for these two reasons, then postgraduates will have a high degree of recognition of the tutor in all directions. They will respect, admire or like the tutor psychologically and are more willing to communicate with the tutor emotionally. Therefore, the Comprehensive Cultivation Interaction between tutors and postgraduates will certainly be more.

The results show that the large number of postgraduates that tutors guide has a significant negative predictive effect on the Comprehensive Cultivation Interaction between tutors and postgraduates, which is a risk factor for high-level Comprehensive Cultivation Interaction between tutors and postgraduates. That is, the greater the number of postgraduates supervised by tutors at the same time, the lower the degree of Comprehensive Cultivation Interaction between tutors and students. The more postgraduates that are instructed by the tutor, the more the tutor’s energy for each postgraduate is inevitably diverted; when the mentor directs the postgraduates with the same energy, less energy will be allocated to each postgraduate, which will lead to the overall degree of interaction between the tutor and the postgraduates being reduced [34].

The results show that the young tutors or senior postgraduates who are not assigned by the tutors for the daily guidance of postgraduates had a significant negative predictive effect on Comprehensive Cultivation Interaction, which is a risk factor for high-level Comprehensive Cultivation Interaction. That is, the mentor appoints young tutors or senior postgraduates to give them daily guidance, which will promote the Comprehensive Cultivation Interaction. This shows that the system of collective guidance is more conducive to the comprehensive cultivation interaction of postgraduate students.

## Related to education practice

Certain practical implications should be considered. The National Education Department should stabilize the enrollment scale of tutors and allocate educational resources accordingly. Colleges and universities need to further strengthen the training of tutors and establish a scientific tutor training system complemented by both professional training and tutor ethics education to ensure the effectiveness of tutors’ teaching behavior and talent training. Universities should guide postgraduates to transform from “tutor-centered” educational ideas to “postgraduates-centered” educational ideas and to build an equal relationship between tutors and postgraduates. The university management department should formulate measures to promote the interaction between tutors and postgraduates according to the actual situation, such as periodic academic seminars, reading reports, or literature reading sessions informed by scientific research groups, and ensuring that tutors and postgraduates meet at regular intervals.

## Limitations

Some limitations must be acknowledged. First, the initial development of the ‘Teacher-Postgraduate Interaction Scale’ for this study requires more cross-cultural testing. Moreover, stratified nonrandom sampling was used for participant recruitment, which may have resulted in a sampling bias and made the sample nonrepresentative of all Chinese postgraduates. Furthermore, although strict quality control was conducted, there may be potential bias in the online cross-sectional survey. In addition, the social approval that the respondents responded to cannot be eliminated.

## Conclusions

The current study enhances the knowledge of the influencing factors in the interaction between tutors and postgraduates in medical universities. The interaction between tutors and postgraduates in medical colleges is generally good, but much attention should be given to the dual-track promotion of the Professional Ability Interaction and Comprehensive Cultivation Interaction. Regular academic seminars play an important role in the process of postgraduate training. The influencing factors were explored regarding the Professional Ability Interaction and Comprehensive Cultivation Interaction. The reasons for postgraduates choosing tutors, the satisfaction of postgraduates with tutors, the satisfaction of postgraduates with learning life, and the higher grade of postgraduates have a positive predictive effect on the interaction between tutors and postgraduates in medical colleges and universities. The older tutors and the greater number of postgraduates that they supervise have a negative predictive effect on the interaction between teachers and students in medical colleges and universities. This study proposes suggestions and strategies for the cultivation of medical talent with high medical technology and ethics from various aspects, such as postgraduate enrollment systems, cultivating among tutors the idea of morality education of students, the improvement of the guidance ability of tutors, and the improvement of the tutor and postgraduate management systems.

## Data Availability

The datasets used and/or analyzed during the current study are available from the corresponding author on reasonable request.
